# Machine Learning Methods for Pregnancy and Childbirth Risk Management

**DOI:** 10.3390/jpm13060975

**Published:** 2023-06-10

**Authors:** Georgy Kopanitsa, Oleg Metsker, Sergey Kovalchuk

**Affiliations:** 1 Faculty of Digital Transformations, ITMO University, 4 Birzhevaya Liniya, 199034 Saint-Petersburg, Russia; 2 Almazov National Medical Research Centre, Ulitsa Akkuratova, 2, 197341 Saint-Petersburg, Russia

**Keywords:** delivery date, childbirth, machine learning, risk factors, prediction

## Abstract

Machine learning methods enable medical systems to automatically generate data-driven decision support models using real-world data inputs, eliminating the need for explicit rule design. In this research, we investigated the application of machine learning methods in healthcare, specifically focusing on pregnancy and childbirth risks. The timely identification of risk factors during early pregnancy, along with risk management, mitigation, prevention, and adherence management, can significantly reduce adverse perinatal outcomes and complications for both mother and child. Given the existing burden on medical professionals, clinical decision support systems (CDSSs) can play a role in risk management. However, these systems require high-quality decision support models based on validated medical data that are also clinically interpretable. To develop models for predicting childbirth risks and due dates, we conducted a retrospective analysis of electronic health records from the perinatal Center of the Almazov Specialized Medical Center in Saint-Petersburg, Russia. The dataset, which was exported from the medical information system, consisted of structured and semi-structured data, encompassing a total of 73,115 lines for 12,989 female patients. Our proposed approach, which includes a detailed analysis of predictive model performance and interpretability, offers numerous opportunities for decision support in perinatal care provision. The high predictive performance achieved by our models ensures precise support for both individual patient care and overall health organization management.

## 1. Introduction

This paper is an extended version of papers presented in the pHealth 2022 and previous pHealth conferences [1,2,3].

The timely identification of risk factors in the early stages of pregnancy, along with effective risk management and mitigation [4], prevention strategies [5], and adherence management [6], have the potential to significantly reduce the occurrence of adverse perinatal outcomes and complications for both mother and child [7]. Considering the existing workload of medical professionals, clinical decision support systems (CDSSs) can play a vital role in assisting with risk management. To ensure the effectiveness of CDSSs, it is essential to develop a robust set of high-quality decision support models that rely on validated medical data and offer clinical interpretability [8].

The development of perinatal episodes involves a complex interplay of numerous heterogeneous factors, each contributing differently to the etiology and pathology at various stages. This complexity poses a significant challenge in developing decision support models. In such a scenario, intelligent data analysis and data-driven models [9] can serve as effective foundations for clinical decision support.

For instance, in a review focusing on risk assessment and management to prevent preterm birth, a study was conducted on 47 patients with connective tissue dysplasia and 29 patients without this syndrome [5]. The study utilized data from clinical and laboratory tests, ultrasound, Dopplerometry, Cardiotocography (CTG), the Electrocardiogram (ECG), and echocardiography (ECHO-CG). By analyzing categorical variables related to the history, course, and outcome of pregnancy, the effects of connective tissue dysplasia were evaluated in a sample of 400 pregnant women. The final dataset consisted of 350 features, and the developed model successfully predicted the probability of complications during pregnancy and childbirth.

The forecast generated by the model accurately predicted complications for 32 out of 50 women, with 16 women having more predicted complications and 3 women having fewer predicted complications. Among the patients, approximately 51% experienced complications, with 86% exhibiting chronic fetal hypoxia and 6% experiencing premature detachment of the normally located placenta. These findings align with the results of previous studies [10,11].

The Apgar score serves as a reliable and widely accepted metric for assessing childbirth outcomes due to its comprehensive evaluation of vital signs, including heart rate, respiration, muscle tone, reflex irritability, and color, providing valuable insights into the immediate well-being and overall health of the newborn [12].

Regarding specific metrics, a study utilizing a novel machine learning algorithm [13] aimed to identify clinically significant predictors of neurocognitive development in newborns with perinatal human immunodeficiency virus (HIV). Through multifactor regression with gradient boosting and fivefold cross-validation, the study successfully identified the predictors that have the greatest impact on the neurocognitive stability of newborns. Another study [14] demonstrated the high accuracy of logistic regression models in predicting neonatal mortality. Furthermore, machine learning algorithms were compared with traditional methods for early assessment of adverse risks in pregnant women [15].

In addition to examining individual studies, recent systematic reviews [15,16,17,18,19,20] have highlighted the limitations of existing models and algorithms in supporting decision-making, particularly in critical situations. The classification and prognosis precision of these models does not exceed 82%, which is considered unsatisfactory. This is primarily attributed to the lack of structured patient data, making it challenging to construct sufficiently accurate mathematical models for pregnancy development. However, the application of machine learning methods has shown promising results in efficient due date prediction based on ultrasound data [21], and artificial neural networks have demonstrated high accuracy in predicting due dates [22]. Thus, despite the experience gained in developing decision-making models and forecasting maternal risks, there is still room for improvement in these models. The further development of such models holds the potential to reduce complications and mortality rates during pregnancy and childbirth.

The goal of this study is to develop real-world-evidence data-driven models based on semi-structured data for pregnancy and childbirth risks prediction. To achieve this goal, we apply machine learning methods to perform a detailed analysis of the importance of predictors for the due date and outcomes to cover the wellbeing of both the mother and children. In this study, we search for the most reliable predictors and identify relationships among them.

## 2. Materials and Methods

We conducted a retrospective analysis of electronic health records from the perinatal Center of the Almazov specialized medical center in Saint-Petersburg, Russia. The dataset was obtained by exporting data from the medical information system. Dataset A consisted of structured and semi-structured data, comprising a total of 73,115 lines corresponding to 12,989 female patients. This dataset covered the period from 1 January 2015 to 31 December 2019. Additionally, Dataset B included 103,414 lines representing 15,681 newly born patients. Each line in the datasets corresponded to a doctor encounter. To combine the data from the two different health information systems, we used the mother identifier.

### 2.1. Data Preparation and Preprocessing

In our study, we obtained a substantial dataset, consisting of 73,115 lines, from Dataset A. This dataset encompassed a wide range of information, including 97 structured features and unstructured arrays of additional medical data. Notably, the unstructured data comprised valuable insights from sources such as the electronic health records (EHRs), specifically the mother anamnesis.

The inclusion of unstructured data from the EHR holds significant importance in capturing comprehensive medical information. These unaltered and unanalyzed data directly reflect the recorded details from the healthcare providers, ensuring the authenticity and integrity of the information. By incorporating the unstructured data alongside the structured features, our dataset becomes more comprehensive and allows for a more comprehensive analysis of the perinatal care context.

**Figure 1:**
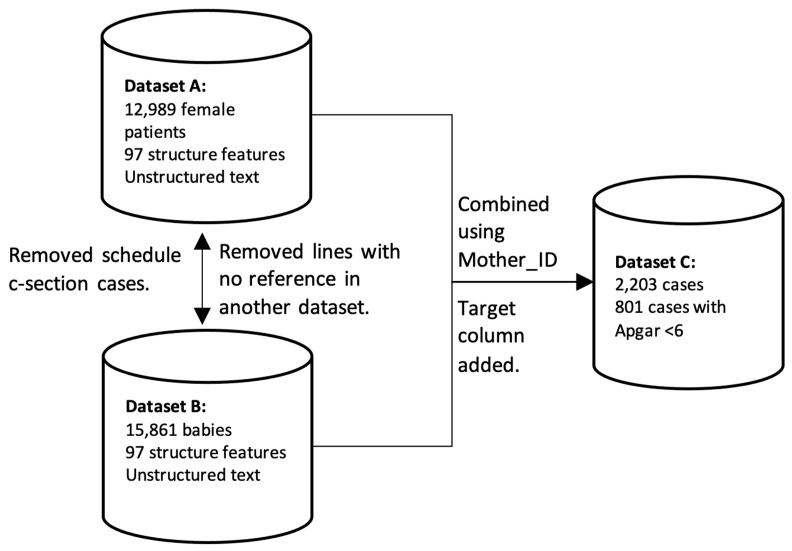
Data preparation process.

Figure 1 visually depicts the data flow and acquisition process, illustrating how the unstructured data from the electronic health records were directly integrated into our dataset. The unmodified inclusion of these data ensures that we capture the most accurate and up-to-date information available, enhancing the overall validity and reliability of our analysis.

The data were taken after the first mandatory screening that takes place between week 11 and 13.

- Mother ID (mother_id) was used as the index to combine two datasets (mothers and newly born).
- All the records from Dataset B that did not have a corresponding mother ID from Dataset A were removed.
- All the lines from Dataset A with no corresponding IDs from Dataset B were removed.
- All the lines that did not contain an Apgar score were removed from the dataset as they were irrelevant for the study.
- All the cases of scheduled C-sections were removed from the datasets.

This resulted in the creation of Dataset C, which comprised 2203 records representing 2203 cases involving both the mother and child. Out of these cases, 801 were identified as having an Apgar score below 6. Any lines in the dataset that did not include the labor date were removed, resulting in 62,734 remaining lines representing an equivalent number of female patients. The target column in the dataset was defined as the length of gestation in days. Additionally, we utilized the Apgar score, ranging from 0 to 10, as a metric for assessing childbirth outcomes [12]. A score of 5 and less was considered as a negative outcome. A target column was added to the dataset: 1 if Apgar score > 5 and 0 if Apgar score < 6.

### 2.2. Correlation and Feature Importance

In our study, we conducted a correlation analysis to explore the relationships between the predictors and the predicted outcomes. To perform this analysis, we employed the Shapley additive explanations (SHAP) index [23], which is a powerful tool for quantifying the contribution and importance of each feature in the prediction model. The SHAP index provides a valuable insight into the role played by each feature in influencing the predictions made by the model. By quantifying the contribution of individual features, it allows us to identify the most relevant predictors that have a significant impact on the model’s predictions.

### 2.3. Prediction Modeling

#### 2.3.1. Childbirth Risks

In order to effectively classify cases with an Apgar score below 6, which serves as an important indicator of potential health risks in newborns, we designed and conducted an experiment. The foundation of our study was Dataset C, a comprehensive collection of relevant information. To ensure the validity and reliability of our findings, we performed a random split of Dataset C, creating a 70% training set and a 30% test set. For the classification task at hand, we opted to employ the random forest (RF) method, a widely recognized and powerful algorithm in the field of machine learning. The RF method operates by constructing an ensemble of decision tree classifiers, each trained on a distinct subset of the dataset. By utilizing this ensemble approach and leveraging the concept of averaging, RF significantly enhances the predictive accuracy of our classification model while effectively mitigating the risk of overfitting.

#### 2.3.2. Due Date Prediction

Each experiment with Dataset C ran in the setting of stratified 5-fold cross-validation, i.e., a random 70% portion of the training dataset was used for training and a random 30% portion of the training dataset was used for testing (70% random selection from the study dataset). Target class ratios in the folds were preserved. The gradient search parameters were: params = {‘min_child_weight’:[4,5], ‘gamma’:[i/10.0 for i in range(3,6)], ‘subsample’:[i/10.0 for i in range(6,11)], ‘colsample_bytree’:[i/10.0 for i in range(6,11)], ‘max_depth’:[2,3,4]}. We compared Gradient Boosting regression, Random forest regression, Linear regression, and Voting regression. The root-mean-square error was used as a performance metric. After determining the optimal dataset and model parameters, we performed a validation with the testing dataset (30% random selection from the study dataset). The Scikit-learn library was used for the experiment. The Mean Absolute Error (MAE) was used as a performance metric. The best performing regressor was evaluated on the test dataset (30% random selection from the study dataset). For this study, we used Python 3.6.3 and scikit-learn 0.19.1 (https://scikit-learn.org/stable/ accessed date: 9.06.2023) as the basic framework for machine learning models.

#### 2.3.3. Model Evaluation

In our experimental analysis, we evaluated the performance of our model on test datasets, which comprised 30% randomly selected lines from the original dataset. We used commonly used performance metrics, including *Precision*, *Recall*, and *F-measure*, to assess the effectiveness of our model.

*Precision* measures the accuracy of positive predictions by calculating the proportion of correctly predicted positive instances out of all instances predicted as positive. It indicates the model’s ability to minimize false positives.

$$Precision=\frac{true\;positives}{true\;positives+false\;positives}$$

*Recall* quantifies the model’s ability to capture positive instances by calculating the proportion of correctly predicted positive instances out of all actual positive instances. It focuses on minimizing false negatives.

$$Recall=\frac{true\;positives}{true\;positives+false\;negatives}$$

*F-measure*, the harmonic mean of precision and recall, provides a balanced assessment of the model’s performance. It considers both false positives and false negatives, offering a comprehensive evaluation.

$$F-measure=2\cdot \frac{recall\cdot precision}{recall+precision}$$

By calculating *Precision*, *Recall*, and *F-measure* on the test datasets, we gain a holistic understanding of our model’s effectiveness in accurately identifying positive instances. These metrics allow us to assess precision, recall rates, and the balance between false positives and false negatives.

## 3. Results

### 3.1. Due Date Prediction

**Figure 2:**
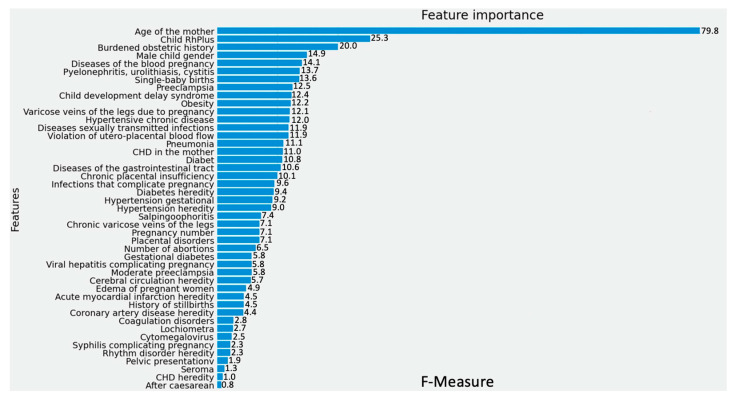
Feature importance for the due date prediction.

This section presents predictors (Figure 2) that include well-known factors such as the mother’s age, as well as previously less explored predictors such as the child’s gender, RH factor, and gastrointestinal diseases. The importance analysis of these features is depicted in Figure 2.

**Figure 3:**
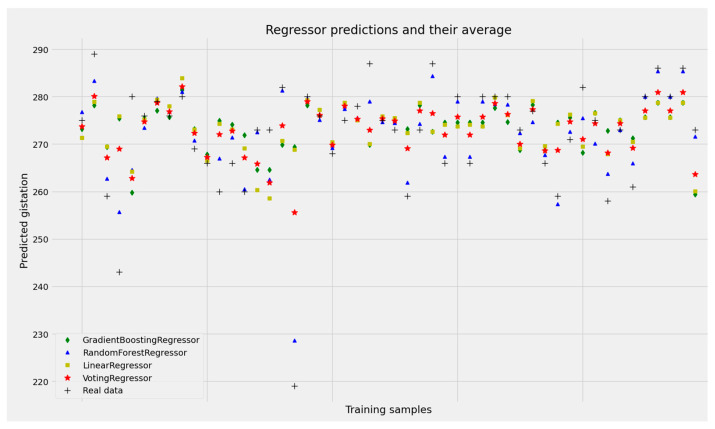
Due date Regression prediction.

Figure 3 and Table 1 present the results of the grid search conducted to find the optimal regression model for due date prediction.

**Figure 4:**
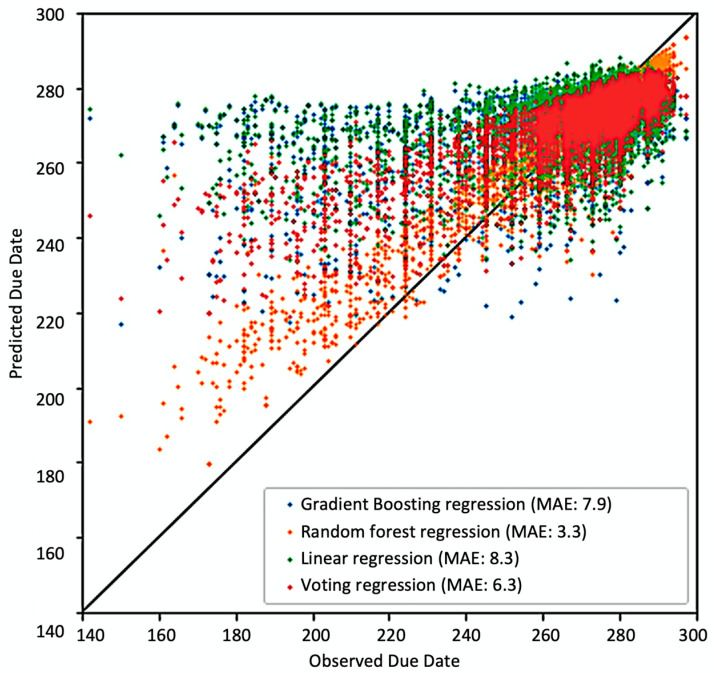
Due date prediction biplot.

Figure 4 presents a due date prediction biplot for different regressors used in the study.

The grid search resulted in the optimal grid parameters: {‘colsample_bytree’:0.9, ‘gamma’:0.3, ‘max_depth’:2, ‘min_child_weight’:4, ‘subsample’:1.0}. We used the MAE for the delivery due date accuracy assessment. The random forest regression gave the best value of MAE of 3.85 on the test dataset.

### 3.2. Childbirth Risk Prediction

#### Correlation and Feature Importance

**Figure 5:**
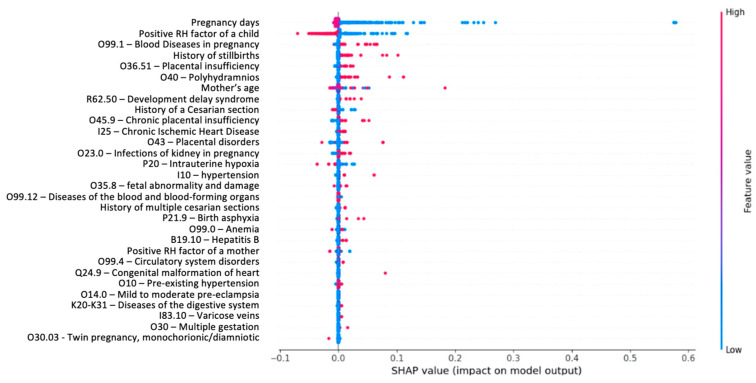
Feature importance for the low APGAR score.

Top important features for Apgar score < 6 are presented in Figure 5.

**Figure 6:**
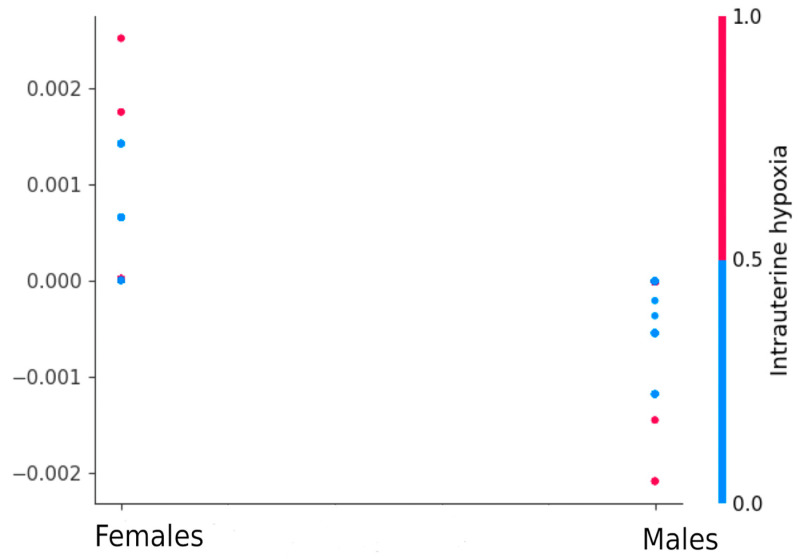
Influence of gender on intrauterine hypoxia.

Figure 6 demonstrates that hypoxia has differential contributions to the risk of low Apgar score in boys and girls. Specifically, hypoxia has a lesser impact on the overall risk of negative outcomes in boys compared to girls. Conversely, intrauterine hypoxia in the fetus can result in intrauterine amniotic fluid aspiration, which increases the probability of stillbirth, particularly in boys.

**Figure 7:**
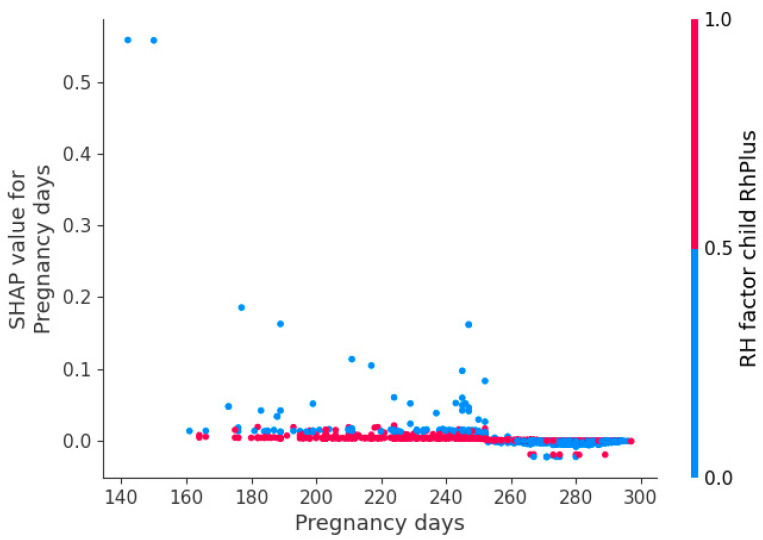
Change in the RH factor influence during the pregnancy.

Figure 7 demonstrates the change in the RH factor influence during the pregnancy.

The Apgar score random forest prediction model achieved a precision of 0.92, indicating a high proportion of correct positive predictions. With a recall of 0.99, it successfully identified the majority of actual positive instances. The F-measure, combining precision and recall, was 0.88, providing an overall assessment of the model’s accuracy.

## 4. Discussion

The findings of our study demonstrate the successful implementation of real-world-evidence data-driven models for the prediction of pregnancy and childbirth risks. By utilizing structured and semi-structured data from electronic health records, this research aimed to develop accurate predictive models that can assist in timely risk identification and improve decision making for medical professionals. We analyzed a comprehensive dataset from a perinatal center, encompassing information from both mothers and newborns, and employed various statistical and machine learning techniques for risk assessment. The results indicate promising outcomes, with the models achieving high precision in predicting adverse childbirth events and due dates. Additionally, the analysis of feature importance revealed clinically significant predictors associated with low Apgar scores, offering valuable insights for early detection and preventive measures. These findings highlight the potential of utilizing data-driven models and real-world evidence to enhance risk management and reduce complications during pregnancy and childbirth.

### 4.1. Clinical Interpretations and Implications

As observed in Figure 6, a low Apgar score is correlated with stillbirth in the medical history, aggravated obstetric history, the mother’s age, presence of uterine scars, and sexually transmitted infections. Complications in the baby are correlated with varicose veins in the legs. Child development delay syndrome is positively correlated with placental insufficiency and fetal growth retardation syndrome, while it is negatively correlated with emergency and spontaneous births.

The male gender of the baby also slightly correlates with newborn complications. Inflammation in the mother can indicate impaired child nutrition, and the development of fetoplacental insufficiency is associated with placental inflammation. Intrauterine intoxication occurs when pyelonephritis affects the kidneys and liver, impairing their function and causing intoxication. Preeclampsia is an indicator that the fetus is suffering, and severe cases may require premature delivery, negatively affecting the fetus. It also disrupts placental blood flow, leading to inadequate nutritional supply. Caesarean sections may be necessary in such cases. Blood diseases, such as anemia, can result in oxygen deficiency and impaired placental oxygen perfusion. When a mother has a blood disease, the child’s circulatory system may suffer from hypoxia as the fetus relies on the placenta for nourishment. The prognosis changes from negative to positive when a premature birth or emergency Caesarean section occurs between 33 and 36 weeks of gestation, as the fetus becomes viable and begins to gain weight. Therefore, it is recommended to exclude cases of emergency Caesarean or premature deliveries when analyzing these factors. Risks for the child should be evaluated separately before and after 33 weeks of gestation.

Complications in the perinatal period should be monitored, and children should be followed up until one year of age with regular monthly check-ups and appropriate tests. Hypertensive disease in the mother triggers a similar mechanism to preeclampsia, leading to oxygen deficiency. This can result in either a Caesarean section or earlier natural delivery. Fetal hypoxia can cause premature labor activity, with the baby experiencing increased breathing and potential asphyxiation from inhaling water, leading to a high heart rate. A mismatch of Rh factors may require intrauterine transfusion, which can result in premature births.

Analysis of the Rh factor indicates changes around the 250th day of pregnancy (see Figure 7). This example highlights the importance of analyzing features in relation to gestational time. Exposure to gastrointestinal diseases in the mother is identified as a significant factor for premature births, despite not typically being considered a risk factor. This finding requires further study. Gastrointestinal diseases may affect the absorption of vitamins and nutrients, possibly due to medications taken for ulcers and gastritis. Obesity disrupts vascular function and leads to metabolic syndrome, hyperglycemia, and plaques in blood vessels. This disturbance in the child’s diet can result in fetoplacental insufficiency and increased labor activity. Varicose disease can have similar consequences. The number of previous abortions and pregnancies in the medical history are obvious factors indicating data accuracy.

### 4.2. Models’ Performance

This study presents the implementation of predictive models for adverse childbirth events, achieving a higher precision (0.92) compared to most state-of-the-art models. The precision of classification and prognosis in previous studies does not exceed 82%, as indicated in the systematic review [9]. The only available models in the literature that performed better were [14] with a precision of 0.93 and [24] with an accuracy of 99.23%. This can be explained in that both studies worked with very limited datasets (285 children and 322 women, respectively).

This is attributed to the inclusion of unstructured medical data alongside the structured dataset. By identifying the main risk factors through feature importance analysis, clinicians can receive support in early complication analysis and the formulation and implementation of preventive measures. The proposed data-driven model for due date prediction enables highly accurate predictions, facilitating effective resource planning. These models are built upon real-world evidence and can be applied with a limited number of predictors. Furthermore, we have identified the most crucial features for predicting the labor due date, aiding policymakers in establishing appropriate data collection channels to capture essential information in electronic health records.

On the other hand, the detailed analysis reveals distinct error patterns in predictive models between the preterm birth period (37 weeks or earlier) and the normal birth period (later than 37 weeks). This discrepancy can be attributed to several factors. Firstly, the dataset exhibits a high level of imbalance, with the majority of cases resulting in normal birth outcomes. Consequently, the models are primarily trained to reflect this normal scenario. Secondly, the nature of preterm birth differs significantly from normal cases, leading to varied performance among different models (although the random forest model still outperforms others). Considering these factors, we believe that dividing the cases based on a rule-based approach or utilizing classifier-based techniques [24,25] and separately training models, with a potential subsequent combination using ensemble techniques [26], could significantly enhance the performance of the model for preterm delivery prediction. We consider addressing this issue as a crucial avenue for future model improvement, given the substantial impact of preterm birth on both maternal and child health, as well as the management of extensive healthcare services.

Even static features should be analyzed in a multifactorial manner rather than through pairwise analysis. Therefore, it is crucial to evaluate the factors influencing labor outcomes from the perspective of the fetus’s gender, as different factors may have contrasting effects on adverse outcomes. Intrauterine hypoxia resulting from intrauterine insufficiency can lead to the aspiration of amniotic fluid, increasing the probability of stillbirth. The intriguing observation that boys are less likely to inhale requires further investigation. Currently, there is limited research that explores the contribution of gender to childbirth outcomes. Our study’s findings (Figure 5 and Figure 6) highlight the need for multifactorial analysis, as opposed to traditional two-factor experiments. The prediction and interpretation of Apgar scores show promising results in improving perinatal health services. Analyzing the performance and interpretation of predictive models reveals similar variations in preterm and normal births. Interpretability plays a vital role in the analysis of predictive models, enabling a deeper understanding of the model structure and its outcomes. Feature engineering is a critical aspect of model development, as it allows mapping features to domain-specific concepts, facilitating more comprehensive interpretation and linking with additional information within patients’ electronic health records. Moreover, such integration enables the incorporation of flexible decision support into existing regulated healthcare processes, promoting greater trust and readiness for predictive models. [27].

The healthcare system generates a vast amount of medical data, comprising both structured and unstructured formats, with unstructured data being predominant. The digital transformation of healthcare necessitates the utilization of all available medical data. The results of this study demonstrate that applying machine learning methods to unstructured data can enhance the accuracy and precision of predictive models. This presents an opportunity to leverage extensive repositories of clinical data for the development of predictive models that aid healthcare professionals in disease diagnosis and recommending appropriate treatment options for patients.

### 4.3. Machine Learning for Clinical Decision Support

In real-time continuous applications, ML methods offer immediate and dynamic decision support, enabling timely risk identification and proactive interventions. This capability is particularly valuable in time-sensitive situations such as emergency obstetric care. ML models continuously analyze data, adapt to changing circumstances, and provide real-time recommendations to enhance perinatal care efficiency. On the other hand, ML methods can also serve as powerful tools for developing decision-making tools that are used in a more static manner. By training on large datasets, ML models capture complex patterns and relationships, offering valuable insights and predictions to guide decision making. The practical incorporation of ML results into accessible decision tools for clinicians is crucial. User-friendly interfaces and visualizations can present the model output in a clear and understandable manner. The collaboration between ML experts and healthcare professionals ensures the development of user-friendly decision support tools that align with clinical needs and workflows.

## 5. Conclusions

The proposed approach offers a range of benefits and opportunities for decision support in perinatal care. One of the key advantages is the high predictive performance achieved by the models. This precision provides valuable support to healthcare services, benefiting both individual patients and health organizations. With accurate predictions, medical professionals can make informed decisions, leading to improved patient outcomes and optimized resource allocation.

Another advantage is the interpretability of the model predictions. This aspect enhances trust and validity, making the technology more suitable for practical use. By understanding the reasoning behind the predictions, healthcare professionals and stakeholders can gain deeper insights into the factors influencing specific outcomes. This transparency fosters trust in the models and facilitates their implementation within clinical settings.

The structured and interpretable nature of the predictive modeling framework also enables further improvements. Researchers can develop even more flexible and interpretable algorithms, expanding the applicability of the approach to diverse scenarios and patient profiles. Additionally, integrating domain-specific knowledge into the models enhances their effectiveness and relevance in perinatal care.

Overall, the results obtained from this study are promising; particularly, they contribute to the overall advancement of a model-based approach with strong predictive performance and clear interpretability. By leveraging these strengths, decision support in perinatal care can be significantly enhanced, leading to improved healthcare outcomes and more efficient resource allocation.

## Figures and Tables

**Table 1:** Prediction efficiency for different regressors.

Regressor	MAE
Random Forrest	3.72
Gradient Boosting	8.02
Linear regression	7.12
Voting regression	6.58

## Data Availability

The datasets GENERATED and ANALYZED for this study can be requested from the corresponding author.
